# Binary Blend All‐Polymer Solar Cells with a Record Efficiency of 17.41% Enabled by Programmed Fluorination Both on Donor and Acceptor Blocks

**DOI:** 10.1002/advs.202202022

**Published:** 2022-06-24

**Authors:** Dehong Zhou, Chentong Liao, Shaoqian Peng, Xiaopeng Xu, Yuanyuan Guo, Jianlong Xia, Huifeng Meng, Liyang Yu, Ruipeng Li, Qiang Peng

**Affiliations:** ^1^ College of Chemistry Key Laboratory of Green Chemistry and Technology of Ministry of Education and State Key Laboratory of Polymer Materials Engineering Sichuan University Chengdu 610065 P. R. China; ^2^ School of Chemical Engineering and State Key Laboratory of Polymer Materials Engineering Sichuan University Chengdu 610065 P. R. China; ^3^ State Key Laboratory of Advanced Technology for Materials Synthesis and Processing Center of Smart Materials and Devices Wuhan University of Technology Wuhan 430070 China; ^4^ Division of Physics and Applied Physics School of Physical and Mathematical Sciences Nanyang Technological University 21 Nanyang Link Singapore 637371 Singapore; ^5^ National Synchrotron Light Source II Brookhaven National Lab Suffolk Upton NY 11973 USA

**Keywords:** all‐polymer solar cells, fluorination, layer‐by‐layer processing, n‐doping, polymer acceptors

## Abstract

Despite remarkable breakthrough made by virtue of “polymerized small‐molecule acceptor (PSMA)” strategy recently, the limited selection pool of high‐performance polymer acceptors and long‐standing challenge in morphology control impede their further developments. Herein, three PSMAs of PYDT‐2F, PYDT‐3F, and PYDT‐4F are developed by introducing different fluorine atoms on the end groups and/or bithiophene spacers to fine‐tune their optoelectronic properties for high‐performance PSMAs. The PSMAs exhibit narrow bandgap and energy levels that match well with PM6 donor. The fluorination promotes the crystallization of the polymer chain for enhanced electron mobility, which is further improved by following *n*‐doping with benzyl viologen additive. Moreover, the miscibility is also improved by introducing more fluorine atoms, which promotes the intermixing with PM6 donor. Among them, PYDT‐3F exhibits well‐balanced high crystallinity and miscibility with PM6 donor; thus, the layer‐by‐layer processed PM6/PYDT‐3F film obtains an optimal nanofibril morphology with submicron length and ≈23 nm width of fibrils, facilitating the charge separation and transport. The resulting PM6/PYDT‐3F devices realizes a record high power conversion efficiency (PCE) of 17.41% and fill factor of 77.01%, higher than the PM6/PYDT‐2F (PCE = 16.25%) and PM6/PYDT‐4F (PCE = 16.77%) devices.

## Graphical abstract

A series of polymerized small molecular acceptors are developed by rational fluorination on the end groups and *π*‐spacers to fine‐tune the photoelectronic properties, molecular crystallinity, and miscibility with the polymer donor, which enables a record power conversion efficiency of 17.41% in layer‐by‐layer processed all‐polymer solar cells.

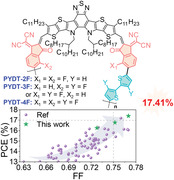

## 1 Introduction

All‐polymer solar cells (all‐PSCs) consisting of a binary blend of a polymer donor and a polymer acceptor possess great potential for being integrated into wearable and portable electronics because of their unique superiorities in high morphological stability, mechanical flexibility, and stress robustness.^[^ ^1^, ^2^, ^3^, ^4^ ^]^ However, compared to the small molecular acceptor (SMA) based polymer solar cells (PSCs) that have occupied the leading position for achieving high power conversion efficiencies (PCEs) over 18%,^[^ ^5^, ^6^, ^7^, ^8^, ^9^, ^10^ ^]^ the development of all‐PSCs lags far behind. This is mainly due to the limited selection pool of high‐performance polymer acceptors relative to the diversity of SMAs.^[^ ^11^, ^12^, ^13^ ^]^ Thanks to the strategy of polymerizing SMA segment into polymer backbone to develop new polymer acceptors,^[^ ^11^ ^]^ a great breakthrough in photovoltaic performances of all‐PSCs has been witnessed. Inspired by the crucial benefits of Y‐series SMAs, such as strong absorption reaching the near infrared (NIR) regions, easily tunable energy levels, and finely tunable crystallinity for realizing high efficiencies with low energy loss (*E*_loss_),^[^ ^14^, ^15^ ^]^ polymerizing them represents a promising way to develop high‐performance polymerized small‐molecule acceptors (PSMAs) that preserve the merits of Y‐series SMAs and has drawn increasing attention in recent years.^[^ ^4^ ^]^ A summary of the‐state‐of‐the‐art Y‐series PSMAs are list in Table S1, Supporting Information. As can be seen, the state‐of‐the‐art PCEs of all‐PSCs based on the Y‐series PSMAs have exceeded 16%,^[^ ^13^, ^16^, ^17^, ^18^, ^19^, ^20^, ^21^, ^22^, ^23^, ^24^ ^]^ and there still has room for further development.

To develop efficient Y‐series PSMAs, great efforts have been made in modifying the donor‐acceptor‐donor (DAD) cores,^[^ ^20^, ^21^, ^22^, ^25^, ^26^ ^]^ end groups,^[^ ^22^, ^27^, ^28^, ^29^, ^30^ ^]^ and *π*‐conjugated spacers.^[^ ^13^, ^31^, ^32^, ^33^, ^34^, ^35^, ^36^, ^37^, ^38^, ^39^ ^]^ In this field, the pioneering efforts of developing Y‐series PSMAs were reported by Min and Huang et al., who employed the Y5 derivatives as the key building blocks and thiophene as the *π*‐conjugated spacer and developed PYT and PJ1, respectively.^[^ ^40^, ^41^ ^]^ The high absorption strength, narrow bandgap (around 1.4 eV), efficient charge separation, and collection enabled their decent short current densities (*J*_sc_s, 21–22 mA cm^−2^) and high PCEs (13–14%). Considering that the Y6 derivatives have narrower bandgaps around 1.3 eV and higher *J*_sc_s (> 25 mA cm^−2^),^[^ ^42^ ^]^ it is essential to broaden the absorption of these PSMAs to further improve their performances. A universal method of broadening the absorption of semiconductors is enhancing the intramolecular charge transfer (ICT) effect by improving the electron donating ability of the electron donor block and/or the electron withdrawing ability of the electron acceptor block. To improve the electron donating ability, the benzothiadiazole (BT) block was replaced with benzotriazole (BTA),^[^ ^20^, ^26^ ^]^ and the thiophene rings were replaced with selenophene rings^[^ ^21^, ^22^, ^25^ ^]^ in the dithienothiopheno[3,2‐b]‐pyrrolobenzothiadiazole (BTP) core. However, the highest occupied molecular orbital (HOMO) levels were upshifted, making these PSMAs could only pair with those donor polymers with high‐lying HOMO levels (i.e., PBDB‐T).^[^ ^20^, ^21^, ^22^, ^25^, ^26^ ^]^ Alternatively, increasing the electron accepting ability of the 1,1‐dicyanomethylene‐3‐indanone (IC) end group by fluorination or chlorination could not only enhance the ICT effect for enhanced absorption but also guaranty low‐lying HOMO levels of these PSMAs to pair with those low‐lying HOMO polymers (i.e., PM6) for realizing better efficiencies.^[^ ^22^, ^27^, ^28^, ^29^, ^43^ ^]^ The *π*‐conjugated spacers were found to have less effect on the band gaps, whereas they could fine‐tune the energy levels and electron transport properties of these PSMAs.^[^ ^20^, ^25^, ^30^, ^31^, ^32^, ^33^, ^34^, ^44^, ^45^ ^]^ Compared with the electron‐rich spacers, the electron‐deficient spacers endowed the PSMAs with lower‐lying energy levels and enhanced electron mobility without sacrificing open‐circuit voltage (*V*_oc_) of the related all‐PSCs.^[^ ^37^, ^39^, ^46^ ^]^ In addition, more and more recent studies have demonstrated that the regioregular PSMAs could obtain better solid‐state packing, synthetic repeatability, and less energy disorder, eventually leading to enhanced charge transport properties and device performances.^[^ ^17^, ^19^, ^26^, ^34^, ^43^, ^45^, ^46^, ^47^, ^48^ ^]^ Therefore, it is essential to take comprehensive consideration of the DAD cores, end groups, *π*‐spacers, and the regiochemistry effect to develop PSMAs toward higher performance.

**Figure 1:**
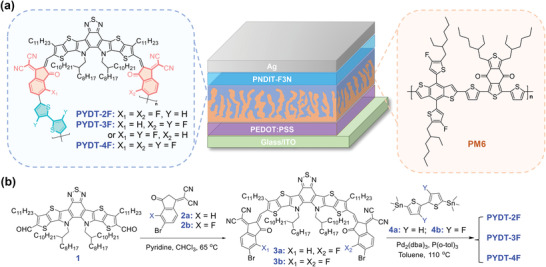
a) Schematic illustration of the device structure of the layer‐by‐layer processed all‐PSCs and the chemical structures of the newly developed PSMAs and PM6 donor. b) Synthetic routes of the newly developed PSMAs.

Herein, we develop a series of PSMAs, named as PYDT‐2F, PYDT‐3F, and PYDT‐4F, by introducing fluorine atoms on the IC groups or/and bithiophene spacers to fine‐tune their optoelectronic properties for high‐performance PSMAs (**Figure** **1**a). It has been reported that the bithiophene spacer could induce weaker aggregation ability of the PSMA and better miscibility with polymer donor than the commonly used thiophene spacer.^[^ ^36^ ^]^ It is well known that the morphology optimization is a long‐standing challenge of all‐PSCs, because the reduced entropic contributions of the PSMAs versus that of SMAs significantly suppress miscibility of polymer donor and acceptor pairs, which often result in severe phase‐separated morphology.^[^ ^2^, ^41^, ^49^, ^50^ ^]^ In this regard, the enhanced miscibility by bithiophene spacers in this work is expected to optimize the morphology of the active layer. On the other hand, the molecular aggregation and charge transport properties can be fine‐tuned by changing the position and number of the fluorine atoms as well as *n*‐doping of the PSMAs. What is more, poor morphology due to unfavorable miscibility can be circumvented by layer‐by‐layer processing, in which the aggregation of polymer donor and acceptor can be optimized separately for realizing more ideal vertical phase separation.^[^ ^51^, ^52^ ^]^ As a result, a record PCE of 17.41% with a high fill factor (FF) of 77.01% was realized for PM6/PYDT‐3F based devices, higher than the PM6/PYDT‐2F (PCE = 16.25%) and PM6/PYDT‐4F (PCE = 16.77%) devices.

## 2 Results and Discussion

### 2.1 Materials Synthesis and Characterization

As illustrated in Figure 1b, Knoevenagel condensation of compound **1** with compounds **2a** and **2b** in one‐pot afforded the asymmetrical compound **3a**, and Knoevenagel condensation of compound **1** with compound **2b** gave the symmetrical compound **3b** in good yield. The target polymer PYDT‐3F was synthesized by Stille‐coupling of **3a** with **4b**, while PYDT‐2F and PYDT‐4F were obtained by Stille‐coupling of **3b** with **4a** and **4b**, respectively. The synthetic details were provided in Supporting Information. The molecular structures were confirmed by nuclear magnetic resonance (NMR) spectra and mass spectra. All three PSMAs show good solubility in common solvents, such as chloroform (CF) and tetrahydrofuran (THF). As determined by gel permeation chromatography with CF as the eluent, the number‐average molecular weight (*M*_n_) and polydispersity index (*Ð*) were measured as 10.9 kDa/1.59 for PYDT‐2F, 12.1 kDa/1.81 for PYDT‐3F, and 11.4 kDa/1.58 for PYDT‐4F, respectively (**Table** **1**). The comparable molecular weights can help to highlight the fluorination effects on the overall properties. All three PSMAs have good thermal stability with decomposition temperatures (*T*_d_, 5% weight loss) over 340 °C (Figure S1, Supporting Information).

**Table 1:** Molecular weight and optical and electrochemical properties of the PSMAs

	*M*_n_ [kDa]	*Ð*	*λ*_max, sol_ [nm]	*ε* [M^−1^ cm^−1^]	*λ*_max, film_ [nm]	*E*_g_^opt^ [eV]	HOMO [eV]	LUMO [eV]
PYDT‐2F	10.9	1.59	767	1.02 × 10^5^	794	1.38	−5.65	−3.90
PYDT‐3F	12.1	1.81	774	1.05 × 10^5^	795	1.37	−5.67	−3.94
PYDT‐4F	11.4	1.58	776	1.08 × 10^5^	801	1.36	−5.68	−3.96

### 2.2 Optical Properties and Energy Levels

**Figure 2:**
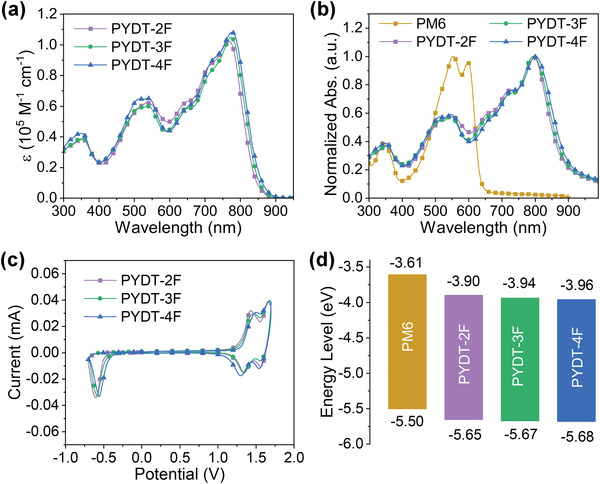
Molar extinction coefficient of the polymer acceptors in chloroform. b) Normalized UV–vis–NIR absorption spectra of the polymer acceptors and PM6 donor in film state. c) CV curves of the polymer acceptors. d) Energy level diagram of the polymer acceptors and PM6 donor.

The UV–vis–NIR absorption spectra of these PSMAs in diluted CF solutions and as films are shown in **Figures** **2**a and **2**b, respectively. Owing to their same conjugated backbones, these PSMAs showed similar absorption profiles in both solution and film state. With increasing the number of fluorine atom, the maximum absorption peak red‐shifted gradually with slightly increased molar extinction coefficient from 767 nm (*ε* = 1.02 × 10^5^ M^−1^ cm^−1^) of PYDT‐2F to 774 nm (*ε* = 1.05 × 10^5^ M^−1^ cm^−1^) of PYDT‐3F and 776 nm (*ε* = 1.08 × 10^5^ M^−1^ cm^−1^) of PYDT‐4F (Table 1), which indicated the enhanced ICT by the fluorine atoms. Going from solutions to films, an obvious red‐shifted absorption (> 20 nm) was observed for all three PSMAs, implying the enhanced molecular packing feature. The optical bandgaps (*E*_g_^opt^s) were determined to be 1.38 eV for PYDT‐2F, 1.37 eV for PYDT‐3F, and 1.36 eV for PYDT‐4F, respectively, based on their absorption onsets. These results indicated the fluorination on the IC groups and bithiophene spacers could somewhat enhance the absorption and narrow the *E*_g_^opt^s of these PSMAs. All three PSMAs exhibited good complementary absorption with the wide bandgap polymer donors, such as PM6 (Figure 2b).

The frontier orbital levels of these PSMAs were measured by cyclic voltammetry (CV) experiments (Figure 2c). The HOMO and lowest unoccupied molecular orbital (LUMO) levels were estimated to be −5.65/−3.90 eV for PYDT‐2F, −5.67/−3.94 eV for PYDT‐3F, and −5.68/−3.96 eV for PYDT‐4F, respectively (Figure 2d and Table 1). These results demonstrated that the energy levels of the PSMAs could be fine‐tuned by varying the number of fluorine atoms. These PSMAs could match well with PM6 (HOMO/LUMO = −5.50/−3.61 eV),^[^ ^53^ ^]^ the small HOMO offsets (*∆E*_HOMO_ < 0.2 eV) were conducive to realize small energy loss in the related all‐PSCs. Density functional theory (DFT) simulations were used to study the effect of fluorination on the molecular geometry and electronic properties (Figure S2, Supporting Information). These PSMAs displayed almost similar molecular geometries, the HOMO/LUMO levels were calculated to be −5.45/−3.43 eV for PYDT‐2F, −5.52/−3.50 eV for PYDT‐3F, and −5.53/−3.52 eV for PYDT‐4F, respectively. These results indicated the fluorination on the IC groups and bithiophene spacers had less effect on the molecular geometry and bandgap, but resulted in lowered energy levels of these PSMAs.

### 2.3 Photovoltaic Properties

The photovoltaic properties of these PSMAs were investigated by fabricating the all‐PSCs with a conventional structure of indium tin oxide (ITO)/poly(3,4‐ethylenedioxythiophene):poly(styrenesulfonate) (PEDOT:PSS)/active layer/PNDIT‐F3N/Ag. PM6 was used as the electron donor, and the layer‐by‐layer processing of the polymer donor (11 mg mL^−1^ in chlorobenzene) and these PSMAs (8 mg mL^−1^ in chloroform with 2 v% 1‐chloronaphthalene as the additive) formed the active layer, which had been demonstrated to be an effective strategy for morphology optimization of all‐PSCs recently.^[^ ^51^, ^52^ ^]^ The details of the device fabrication procedure were presented in Supporting Information.

**Figure 3:**
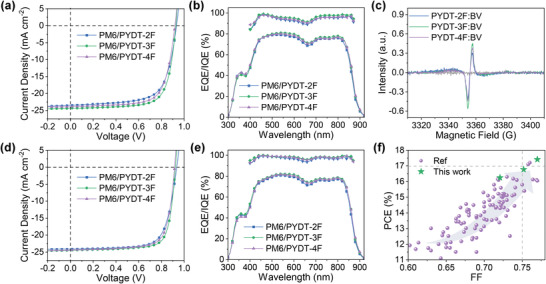
a) *J–V* curves of the all‐PSCs without BV doping. b) EQE and IQE spectra of the all‐PSCs without BV doping. c) ESR spectra of the PSMAs doped with 0.04 wt% BV. d) *J–V* curves of the all‐PSCs doped with 0.04 wt% BV. e) EQE and IQE spectra of the all‐PSCs doped with 0.04 wt% BV. f) The summarized PCE versus FF plots of the all‐PSCs based on Y‐series PSMAs.

As shown in **Figure** **3**a and **Table** **2**, the PYDT‐2F‐based devices achieved a high *V*_oc_ of 0.933 V, but a moderate *J*_sc_ of 23.56 mA cm^−2^ and FF of 68.26%, limiting the PCE to 15.00%. In comparison, PYDT‐3F‐based devices exhibited slightly lower *V*_oc_ of 0.927 V because of the reduced LUMO level of PYDT‐3F. Nevertheless, the significantly enhanced *J*_sc_ of 24.25 mA cm^−2^ and FF of 72.86% contributed to a higher PCE of 16.38%. With introducing more fluorine atoms, the PYDT‐4F‐based devices exhibited simultaneously reduced *V*_oc_ of 0.918 V, *J*_sc_ of 23.89 mA cm^−2^, and FF of 71.32%, resulting in the lowered PCE of 15.56%. The fluorination effect on the photovoltaic performances was further studied by the external quantum efficiency (EQE) and internal quantum efficiency (IQE) measurement (Figure 3b and Figure S3, Supporting Information). These all‐PSCs exhibited high EQE responses in two main ranges of 300–700 and 700–900 nm, which contributed from the PM6 donor and the PSMAs, respectively. The PYDT‐3F and PYDT‐4F‐based devices displayed higher EQE response than PYDT‐2F‐based devices, indicating their higher charge extraction and/or collection efficiencies. The current density integrated from the EQE spectra (*J*_EQE_) was 23.12 mA cm^−2^ for PYDT‐2F, 23.76 mA cm^−2^ for PYDT‐3F, and 23.43 mA cm^−2^ for PYDT‐4F‐based devices, respectively, which were quite closed to the *J*_sc_ values obtained from the current density–voltage (*J–V*) curves. As these PM6/PSMA films exhibited almost comparable absorption in the devices (Figure S3, Supporting Information), the difference of their current densities should be attributed to their different IQE responses that are affected by the charge generation and recombination process.

**Table 2:** Photovoltaic parameters of the devices containing different PSMAs without or with BV doping

D/A	BV doping	*V*_oc_ [V]	*J*_sc_ [mA cm^−2^]	*J*_EQE_ [mA cm^−2^]	FF [%]	PCE [%]
PM6/PYDT‐2F	No	0.933	23.56	23.12	68.26	15.00 (14.74 ± 0.39)^a)^
PM6/PYDT‐3F	No	0.927	24.25	23.76	72.86	16.38 (16.07 ± 0.22)
PM6/PYDT‐4F	No	0.918	23.89	23.43	71.32	15.72 (15.48 ± 0.16)
PM6/PYDT‐2F	Yes	0.935	24.11	23.62	72.08	16.25 (15.82 ± 0.30)
PM6/PYDT‐3F	Yes	0.923	24.49	24.26	77.01	17.41 (17.10 ± 0.18)
PM6/PYDT‐4F	Yes	0.915	24.37	24.01	75.20	16.77 (16.51 ± 0.21)

^a)^ Average PCEs with standard deviations were calculated from ten individual devices.

The molecular doping has been demonstrated to be an efficient method to promote the charge transport of donor and acceptor materials in both SMA‐based PSCs and all‐PSCs to improve their photovoltaic performances.^[^ ^54^, ^55^ ^]^ Inspired by these, we employed benzyl viologen (BV) as a dopant to n‐dope the PSMAs and tried to further improve the device performance of these all‐PSCs. As expected, all three PSMAs exhibited obvious electron spin resonance (ESR) signals, which confirmed that these PSMAs had been *n*‐doped by BV additive (Figure 3c). The BV doping effect on the electron transport properties of the PSMAs was studied by using the space charge limited current (SCLC) method (Figure S4, Supporting Information). The electron mobility (*µ*_e_) of pristine PYDT‐2F was estimated to be 2.40 × 10^−4^ cm^2^ V^−1^ s^−1^. Compared with PYDT‐2F, PYDT‐3F and PYDT‐4F showed higher *µ*_e_ values of 5.02 × 10^−4^, and 5.54 × 10^−4^ cm^2^ V^−1^ s^−1^, respectively. The higher *µ*_e_ of PYDT‐3F and PYDT‐4F were conducive to more efficient charge transport within the active blends for higher device performances. After being doped with BV, the *µ*_e_ values were increased to 5.25 × 10^−4^, 9.71 × 10^−4^, and 1.07 × 10^−3^ cm^2^ V^−1^ s^−1^ for PYDT‐2F, PYDT‐3F, and PYDT‐4F, respectively, which were expected to improve the device performances further.

As shown in Figure 3d and Table 2, the BV doping showed minimal effect on the *V*_oc_s of these all‐PSCs. However, the *J*_sc_ and FF were significantly increased in the presence of BV dopant. The *J*_sc_ and FF were increased to 24.11 mA cm^−2^ and 72.08%, respectively, for PYDT‐2F‐based devices, thus the PCE was improved to 16.25% with a high *V*_oc_ of 0.935 V. Excitingly, PYDT‐3F devices exhibited much higher *J*_sc_ of 24.49 mA cm^−2^ and FF of 77.01%, contributing to a champion PCE of 17.41%. In addition, PYDT‐4F devices also realized a decent PCE of 16.77%, owing to the enhanced *J*_sc_ of 24.37 mA cm^−2^ and FF of 75.20%. The improved device performances by BV doping method were also confirmed by the EQE and IQE experiments, which displayed increased photo response over the absorption range (Figure 3e). The *J*_EQE_s were increased to 23.62, 24.26, and 24.01 mA cm^−2^ for PYDT‐2F, PYDT‐3F, and PYDT‐4F‐based devices, respectively, which agreed well with the *J*_sc_s obtained from the *J–V* curves. The BV doping had minimal effect on the light absorption (Figure S3b, Supporting Information) but significantly improved the IQE of the all‐PSCs, indicating the enhanced charge extraction after doping. Note that the great challenge lying in the morphology control makes it still difficult for achieving a high FF over 75% and a PCE over 17% in all‐PSCs (Figure 3f and Table S1, Supporting Information). In this work, an impressive high FF of 77.01% and a new record PCE of 17.41% were realized finally, which were contributed from the rational fluorination of PSMAs, layer‐by‐layer processing technology, and *n*‐doping process. Compared with the layer‐by‐layer processed devices, the bulk heterojunction (BHJ) devices achieved lower PCEs of 15.69%, 16.67%, and 16.19% for PM6:PYDT‐2F, PM6:PYDT‐3F, and PM6:PYDT‐4F, respectively (Figure S5 and Table S2, Supporting Information). There results indicate the layer‐by‐layer processing is a promising strategy for improving the device performances of all‐PSCs. Using another polymer D18‐Cl as the electron donor in the layer‐by‐layer devices, PYDT‐3F‐based devices could also realize a higher PCE of 16.07% than the devices based on PYDT‐2F (14.31%) and PYDT‐4F (15.40%) (Figure S6 and Table S3, Supporting Information). These results demonstrate that the rational fluorination strategy is universal in developing high performance PSMAs.

### 2.4 Morphology Properties

**Figure 4:**
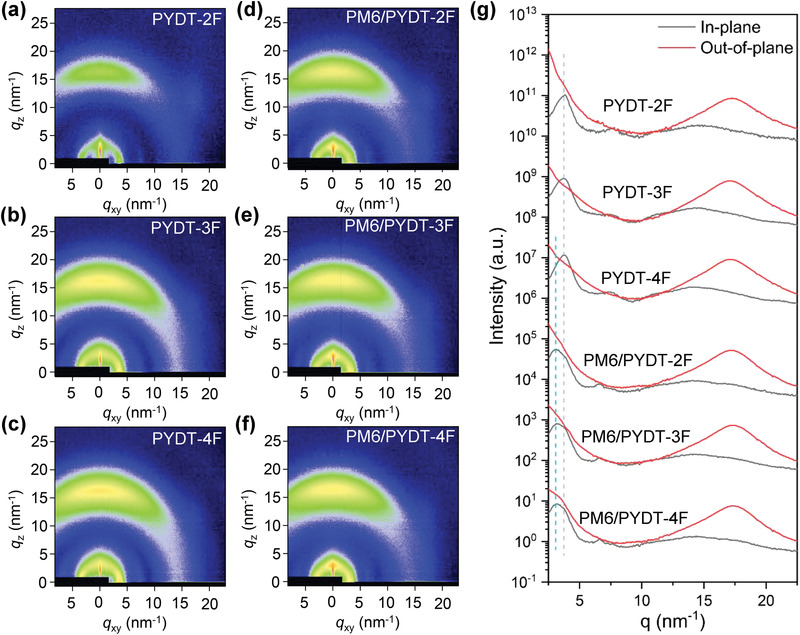
GIWAXS patterns of a–c) the pristine PSMA films and d–f) the PM6/PSMA films. g) The corresponding in‐plane and out‐of‐plane line‐cuts.

To study the molecular packing behavior, grazing incidence wide‐angle X‐ray scattering (GIWAXS) measurements were performed. The PM6 donor showed obvious lamellar diffractions in both the in‐plane and out‐of‐plane directions with q = 3.01 nm^−1^ (lamellar stacking distance, d_l_ = 20.9 Å), indicating the coexistence of edge‐on and face‐on packing (Figure S7 and Table S4, Supporting Information). A well‐defined 010 diffraction was located at q_z_ = 17.4 nm^−1^ in the out‐of‐plane direction, corresponding to the preferred face‐on *π*–*π* stacking with a distance (*d*_*π*_) of 3.61 Å. The PSMAs displayed quite similar stacking behaviors with a preferred face‐on packing, owing to their same molecular backbones (**Figure** **4**). The lamellar stackings were located at around 3.72 – 3.77 nm^−1^ (d_l_ = 16.9 – 16.7 Å) and the *π*–*π* stackings were located at 17.2 nm^−1^ (*d*_*π*_ = 3.65 Å). However, the crystal coherent length (CCL) was increased gradually from 56.1 to 60.1 and 64.5 Å for lamellar stackings, and from 16.0 to 19.9 and 21.3 Å for *π*–*π* stackings, indicating the enhanced crystallinity with introducing more fluorine atoms onto the polymer backbone. The layer‐by‐layer processed PM6/PSMA films showed higher crystallinity than the pristine donor and PSMAs films, which originated from the rearrangement of PM6 donor during the PSMA layer deposition. On the other hand, the presence of 1‐chloronaphthalene (CN) additive also promoted the crystallization of both PM6 and PMSAs. The lamellar diffraction could be divided into two peaks, which were assigned to the PM6 donor and the PMSA, respectively (Figure S8, Supporting Information). Compared to PYDT‐2F (CCL of 62.8 Å for lamellar stacking), the higher CCLs (for lamellar stacking) of 69.8 and 71.5 Å for PYDT‐3F and PYDT‐4F, respectively, indicated their higher but similar crystallinity. These results explained the higher electron mobilities of PYDT‐3F and PYDT‐4F as aforementioned before.

In addition to the crystallinity, the morphology plays a vital role to determine the device performance. To predict the morphology of the PM6/PSMA films, the droplet contact angles were measured (Figure S9, Supporting Information). The surface energy (*γ*) was calculated to be 36.4 mJ m^−2^ for the PM6 donor. PSMAs exhibited higher *γ* values, which were 42.4 mJ m^−2^ for PYDT‐2F, 41.7 mJ m^−2^ for PYDT‐3F, and 40.3 mJ m^−2^ for PYDT‐4F, respectively. The Flory–Huggins parameter (*χ*) between PM6 donor and the PSMAs could be calculated via the equation of $\chi_{12}=\;K{\left(\sqrt{\gamma_{1}}-\sqrt{\gamma_{2}}\right)}^{2}$, where *K* is a proportionality constant.^[^ ^56^ ^]^ The calculated *χ* values were 0.23, 0.18, and 0.10 K for PYDT‐2F, PYDT‐3F, and PYDT‐4F, respectively. The successively reduced *χ* values with the increasing number of fluorine atom on the backbone of these PSMAs indicated that the enhanced miscibility would be expected when mixed with PM6 donor.

**Figure 5:**
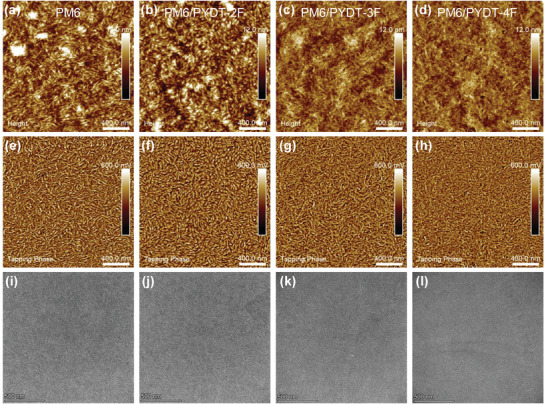
AFM a–d) height images, e–h) tapping phase images, and i–l) TEM images. a,e) Pristine PM6 film. b,f) PM6/PYDT‐2F. c,g) PM6/PYDT‐3F. d,h) PM6/PYDT‐4F.

The fluorination effect on the morphology was further investigated by atomic force microscopy (AFM) (**Figure** **5**a–h). The pristine PM6 film prepared from chlorobenzene solution had a root mean square roughness (RMS) of 1.77 nm, the AFM phase and TEM images displayed a well‐defined nanofibrous net framework. Such a unique nanofiber structure could enable the acceptor molecules fill into the net framework during sequential deposition, forming a pseudo bilayer structure. PM6/PYDT‐2F showed a more needle‐like structure with slightly increased RMS of 1.89 nm. The average width of the nanofibers was estimated to be 36 nm, which is slightly larger than the optimal domain size (10 – 20 nm). The large phase separation between PM6 and PYDT‐2F might be mainly due to their relative poorer miscibility, which promoted the aggregation of PYDT‐2F and reduced the area of the D/A interfaces for charge separation. This explained the reason that PYDT‐2F‐based devices had lower photovoltaic performances. In contrast, the enhanced miscibility made PM6/PYDT‐3F had a more threadlike nanofiber structure with reduced RMS of 1.32 nm. The average fiber width was reduced to 23 nm, facilitating the charge separation and transport. Therefore, PM6/PYDT‐3F‐based devices achieved higher *J*_sc_ and FF values. The further increased miscibility between PM6 and PYDT‐4F led to the smoother PM6/PYDT‐3F film with the smallest RMS of 1.17 nm. The fiber width of PM6/PYDT‐4F (≈21 nm) was quite closed to that of PM6/PYDT‐3F for efficient charge separation. However, the fiber length was largely shortened, which was unfavorable for charge transport, thus resulting in lowered device performances. The fluorination effect on the fiber length and width was also confirmed by the transmission electron microscopy (TEM) measurements (Figure 5i–l). PM6/PYDT‐3F exhibited the mediate fiber length and width for improving the exciton diffusion and charge transport (vide infra). Compared to the layer‐by‐layer processed films, the BHJ films exhibited relative inhomogeneous fiber like morphology (Figure S10, Supporting Information), which explained the lower performances of the BHJ devices.

### 2.5 Charge Generation and Recombination Kinetics

**Figure 6:**
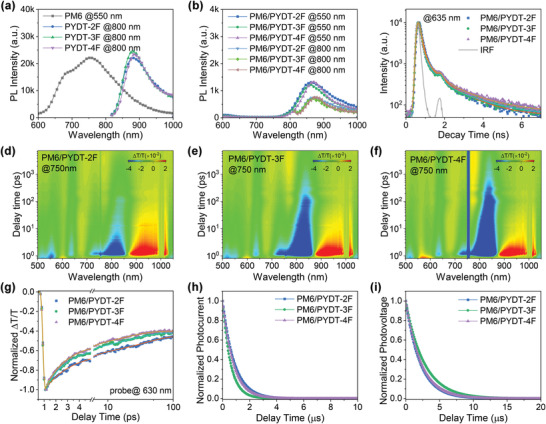
a) PL spectra of the pure films. b) PL spectra of the PM6/PSMA films. c) TRPL of the PM6/PSMA films. d–f) 2D fs‐TAS images of the PM6/PSMA films pumped at 750 nm. g) fs‐TAS decay kinetics of the PM6/PSMA films (pumped at 800 nm, probed at 630 nm). h) TPC of the PM6/PSMA films. i) TPV of the PM6/PSMA films.

The fluorination effect on the charge dissociation process was studied by the steady‐state photoluminescence (PL) (**Figure** **6**a,b) and time‐resolved photoluminescence (TRPL) (Figure 6c) of the all‐PSCs. The PL emissions of both donor (excited at 550 nm) and acceptor (excited at 800 nm) were largely quenched in the PM6/PSMA films, suggesting that the excitons generated in both PM6 and the PSMAs could be dissociated at the D/A interfaces. The PL quenching efficiency of donor emission was calculated to be 95.4% in PM6/PYDT‐2F, 96.4% in PM6/PYDT‐3F, and 96.5% in PM6/PYDT‐4F, respectively. In addition, the PL quenching efficiency of the acceptor was calculated to be 95.1% in PM6/PYDT‐2F, 96.0% in PM6/PYDT‐3F, and 96.0% in PM6/PYDT‐4F, respectively. PM6/PYDT‐3F and PM6/PYDT‐4F exhibited comparable PL quenching efficiency and higher than that of PM6/PYDT‐2F, showing the more efficient exciton dissociation in PM6/PYDT‐3F and PM6/PYDT‐4F films. From the TRPL measurements, the average fluorescence lifetimes (*τ*) were 106 ± 26 ps for PM6/PYDT‐2F, 103 ± 24 ps for PM6/PYDT‐3F, and 102 ± 16 ps for PM6/PYDT‐4F, which were close to the limitation of our instrument response function. The higher PL quenching efficiency and shorter fluorescence lifetime of PM6/PYDT‐3F and PM6/PYDT‐4F correlated with the smaller fiber width of them than that of PM6/PYDT‐2F, which provided more D/A interfaces favoring exciton dissociation.

The charge generation and recombination dynamics in PM6/PSMA films were further investigated by femtosecond transient absorption spectroscopy (fs‐TAS) measurements. The ground‐state bleach (GSB) evolution of PM6 was around 630 nm and the GSB signals of PSMAs were around 800 nm (Figure S11, Supporting Information). PM6 donor could not be selectively excited because the PSMAs also exhibited strong absorption in the PM6 absorption range (Figure 2b). Therefore, an excitation wavelength at 750 nm was chosen to excite the PSMAs to study the electron transfer processes (Figure 6d,e,g). Here, the kinetics of 630 nm was chosen to represent the PM6 GSB dynamic owing to the photoexcited PSMAs displayed almost no signals there. The rising kinetics of PM6 GSB in the PM6/PSMA films directly reflected the hole transfer process, which could be fitted by biexponential function. The hole transfer process consisted an ultrafast hole transfer process at the D/A interface, as characterized by *τ*_1_, as well as a diffusion mediated process controlled by domain size and aggregation strongly, as characterized by *τ*_2_.^[^ ^52^ ^]^ The *τ*_1_/*τ*_2_ were estimated to be 2.1/35.3 ps for PM6/PYDT‐2F, 1.7/20.6 ps for PM6/PYDT‐3F, and 1.6/16.8 ps for PM6/PYDT‐4F, respectively. PM6/PYDT‐2F showed larger *τ*_1_ and *τ*_2_ values than PM6/PYDT‐3F and PM6/PYDT‐4F, which was mainly due to the reduced D/A interfaces that lowered the hole transfer efficiency and larger domains that increased the exciton diffusion time. The slower exciton diffusion and hole transfer could result in the higher exciton losses in PM6/PYDT‐2F devices, which might explain the inferior photovoltaic performances of PM6/PYDT‐2F based devices. PM6/PYDT‐3F showed comparable hole transfer time with PM6/PYDT‐4F, suggesting their high charge generation yield. The extended distance of exciton diffusion in PM6/PYDT‐3F was originated from the slightly large fiber width of PYDT‐3F. Although PM6/PYDT‐4F exhibited the shortest times for exciton diffusion and hole transfer, the corresponding devices did not realize the best photovoltaic performances. A possible reason might be the small domains with excessive D/A interfaces led to high‐rate bimolecular recombination of free charges in the PM6/PYDT‐4F devices.

To directly determine the charge carrier extraction and recombination mechanisms, transient photocurrent (TPC) and transient photovoltage (TPV) techniques were conducted. As shown in Figure 6h, photocurrent decay times were estimated to be 0.83, 0.56, and 0.77 µs for PM6/PYDT‐2F, PM6/PYDT‐3F, and PM6/PYDT‐4F based devices, respectively. The shorter photocurrent decay time suggested a faster charge sweep‐out of PM6/PYDT‐3F based all‐PSCs than the other devices. In addition, photovoltage decay times were estimated to be 2.13, 2.34, and 2.27 µs for PM6/PYDT‐2F, PM6/PYDT‐3F, and PM6/PYDT‐4F based devices, respectively. The longer photovoltage decay time indicated the longer charge carrier lifetime of PM6/PYDT‐3F based all‐PSCs than the other devices. The combination of TPC and TPV results demonstrated the lowest charge recombination rate in PM6/PYDT‐3F devices, which contributed to the best photovoltaic performances.

## 3 Conclusion

In summary, three PSMAs sharing the same backbone, named as PYDT‐2F, PYDT‐3F, and PYDT‐4F, were developed by introducing different fluorine atoms on the IC groups or/and bithiophene spacers to fine‐tune their optoelectronic properties for high‐performance PSMAs. With increasing fluorine numbers on the polymer backbone, the *E*_g_^opt^ was slightly narrowed from 1.38 to 1.36 eV, and the LUMO level was lowered from −3.89 to −3.94 eV. Moreover, the fluorination promoted the crystallization of the polymer chain for enhanced electron mobility, which was further improved by following *n*‐doping with BV additive. Importantly, by using layer‐by‐layer processing, the aggregation of the PM6 donor and these PSMAs could be optimized separately, avoiding the severe phase‐separation and low FF values that frequently observed in all‐PSCs. The surface energy was gradually lowered with increasing the number of fluorine atom on the PSMA backbone, which resulted in the improved miscibility with PM6 donor. The excellent balance in crystallinity and miscibility made PM6/PYDT‐3F achieve optimal nanofibril morphology with submicron length and ≈23 nm width of fibrils, facilitating the charge separation and transport. As a result, a champion PCE of 17.41% with satisfied FF of 77.01% was realized for PM6/PYDT‐3F‐based devices, higher than the PM6/PYDT‐2F‐based devices (PCE = 16.25%, FF = 72.08%) and the PM6/PYDT‐4F‐based devices (PCE = 16.77%, FF = 75.20%). The results demonstrate the combination of chemical modification, *n*‐doping of the Y‐series PSMAs, and layer‐by‐layer processing is promising to optimize the photoelectronic properties and blend morphology for maximizing the device performances of all‐PSCs.

## Conflict of Interest

The authors declare no conflict of interest.

## Supporting information

Supporting Information

## Data Availability

Research data are not shared.
