# Supplementary material for: Binary Blend All‐Polymer Solar Cells with a Record Efficiency of 17.41% Enabled by Programmed Fluorination Both on Donor and Acceptor Blocks
Source: Adv Sci (Weinh). 2022 Jun 24;9(23):2202022. doi: 10.1002/advs.202202022 (PMC9376845; doi:10.1002/advs.202202022)
Supplement: Supplementary file 1 — Supporting Information [file ADVS-9-2202022-s001.pdf]

ADVANCED SCIENCE
Open Access

Supporting Information

for *Adv. Sci.*, DOI 10.1002/advs.202202022

Binary Blend All-Polymer Solar Cells with a Record Efficiency of 17.41% Enabled by Programmed Fluorination Both on Donor and Acceptor Blocks

Dehong Zhou, Chentong Liao, Shaoqian Peng, Xiaopeng Xu, Yuanyuan Guo, Jianlong Xia*, Huifeng Meng, Liyang Yu, Ruipeng Li and Qiang Peng**

Supporting Information

Binary Blend All-Polymer Solar Cells with a Record Efficiency of 17.41% Enabled by Programmed Fluorination Both on Donor and Acceptor Blocks

Dehong Zhou, Chentong Liao, Shaoqian Peng, Xiaopeng Xu, Yuanyuan Guo, Jianlong Xia, Huifeng Meng, Liyang Yu, Ruipeng Li, and Qiang Peng**

1. Materials and methods

Materials: PM6 was purchased Solarmer Materials Inc. D18-Cl, compounds **2a** and **2b** were purchased from was purchased from eFlexPV Limited. Compounds **4a** and **4b** were purchased from Derthon Optoelectronics Materials Science Technology Co., Ltd. Compounds **1** and **3b** were synthesized according to the previous reports.[1,2] All the other chemicals were purchased from Aladdin, Adamas, Sigma-Aldrich, and Alfa Asear Chemical Co., and used without further purification. All solvents were freshly distilled immediately prior to use.

Synthesis of compound 3a: Compound **1** (0.8348 g, 0.6119 mmol), **2a** (0.1846 g, 0.6760 mmol) and **2b** (0.1870 g, 0.6424 mmol) were dissolved in the mixed solvent of chloroform (30 mL) and pyridine (1 mL). The mixture was deoxygenated with nitrogen and then refluxed for 6 h. After cooling to room temperature, the mixture was poured into methanol (500 mL) and filtered. The residue was purified by column chromatography on silica gel using petroleum ether/dichloromethane (1:2) as eluent to afford compound **3a** as a dark blue solid (0.4120 g, 67.33%). ^{1}H NMR (400 MHz, $CDCl_3$) δ 9.00 (s, 2H), 8.54 (t, 1H), 8.40 (t, 1H), 8.05 (s, 1H), 7.91 (t, 1H), 7.85 (t, 1H), 4.81 (s, 4H), 3.19 (s, 4H), 2.18 (s, 2H), 1.93 – 1.80 (m, 4H), 1.46 (t, J = 7.6 Hz, 4H), 1.39 – 0.89 (m, 92H), 0.81 (dq, J = 14.8, 7.5, 7.0 Hz, 18H). ^{13}C NMR (101 MHz, $CDCl_3$) δ 186.72, 184.41， 159.61, 158.69, 154.16, 153.79, 153.59, 147.46, 145.26, 145.14, 135.34, 133.97, 133.47, 130.67, 123.55, 123.42, 121.68, 119.24, 116.62, 116.43, 115.23, 114.90, 113.63, 68.45, 55.57, 39.33, 31.98, 31.95, 31.87, 31.60, 31.18, 30.69, 30.01, 29.91, 29.72, 29.67, 29.60, 29.52, 29.44, 29.39, 29.36, 25.59, 22.71, 22.64, 22.34, 14.12. HR-MS (MALDI-TOF) [M+H]+ calcd. for ($C_{106}H_{135}Br_2F_2N_8O_2S_5$): 1892.4064; found: 1892.6026.

Synthesis of PYDT-2F: Compound **3b** (49.30 mg, 0.0258 mmol), **4a** (12.69 mg, 0.0258 mmol), $Pd_2(dba)_3$ (1.18 mg, 0.00129 mmol), P(*o*-tolyl)$_3$ (1.57 mg, 0.00516 mmol) were

dissolved in and toluene (2 mL). The mixture was deoxygenated with nitrogen and then stirred at 110 ^{o}C for 3 days. After finished, the reaction mixture was cooled down to room temperature and precipitated into methanol (200 mL). The crude polymer was subjected to Soxhlet extractions with methanol, hexane and acetone to remove the impurities and oligomers. Then the chloroform fraction was concentrated and precipitated with methanol to afford PYDT-2F as a purple-black solid. ^{1}H NMR (400 MHz, $CDCl_3$) δ 8.49-8.38 (br, 4H), 7.85-7.44 (br, 6H), 4.95-4.70 (br, 4H), 3.31-3.22 (br, 4H), 2.37-2.44 (m, 2H), 1.94-0.80 (m, 124H).

Synthesis of PYDT-3F: Compound **3b** (48.82 mg, 0.0258 mmol), **4a** (13.62 mg, 0.0258 mmol), $Pd_2(dba)_3$ (1.18 mg, 0.00129 mmol), P(*o*-tolyl)$_3$ (1.57 mg, 0.00516 mmol) were dissolved in and toluene (2 mL). The mixture was deoxygenated with nitrogen and then stirred at 110 ^{o}C for 3 days. After finished, the reaction mixture was cooled down to room temperature and precipitated into methanol (200 mL). The crude polymer was subjected to Soxhlet extractions with methanol, hexane and acetone to remove the impurities and oligomers. Then the chloroform fraction was concentrated and precipitated with methanol to afford PYDT-3F as a purple-black solid. ^{1}H NMR (400 MHz, $CDCl_3$) δ 8.96-8.35 (br, 4H), 8.12-7.46 (br, 5H), 5.01-4.58 (br, 4H), 3.49-2.85 (br, 4H), 2.53-2.28 (m, 2H), 1.94-0.80 (m, 124H).

Synthesis of PYDT-4F: Compound **3b** (49.3 mg, 0.0258 mmol), **4a** (13.62 mg, 0.0258 mmol), $Pd_2(dba)_3$ (1.18 mg, 0.00129 mmol), P(*o*-tolyl)$_3$ (1.57 mg, 0.00516 mmol) were dissolved in and toluene (2 mL). The mixture was deoxygenated with nitrogen and then stirred at 110 ^{o}C for 3 days. After finished, the reaction mixture was cooled down to room temperature and precipitated into methanol (200 mL). The crude polymer was subjected to Soxhlet extractions with methanol, hexane and acetone to remove the impurities and oligomers. Then the chloroform fraction was concentrated and precipitated with methanol to afford PYDT-4F as a purple-black solid. ^{1}H NMR (400 MHz, $CDCl_3$) δ8.96-8.40 (br, 4H), 8.12-7.46 (br, 4H), 5.01-4.59 (br, 4H), 3.51-2.85 (br, 4H), 2.54-2.28 (m, 2H), 1.94-0.80 (m, 124H).

Methods: ^{1}H and ^{13}C NMR spectra were recorded on a Bruker Avance-400 spectrometer with *d*-chloroform as solvent. The chemical shifts were reported as *δ* value (ppm) relative to an internal tetramethylsilane (TMS) standard. Matrix assisted laser desorption ionization time of

flight mass spectrometry (MALDI-TOF-MS) was recorded on a Bruker APEX II Fourier transform ion cyclotron resonance mass spectrometry (FT-ICR-MS) system. Thermogravimetric analysis (TGA) was conducted on a TA Instrument Model SDT Q500 at a heating rate of 10 $^{o}C\ min^{-1}$ and under a N_2 flow rate of 90 mL min^{-1}. UV-vis spectra were obtained on a Hitachi U2910 spectrophotometer. CV measurements were made on a CHI660 potentiostat/galvanostat electrochemical workstation at a scan rate of 50 mV s^{-1}. GIWAXS measurements were performed at Complex Materials Scattering (CMS) beamline of the National Synchrotron Light Source II (NSLS-II), Brookhaven National Lab. Droplet contact angle was measured on a Krüss DSA100 contact angle meter. AFM images were obtained by using a Bruker Inova atomic microscope in tapping mode. TEM images were obtained by a Hitachi HT7700 transmission electron microscope. Femtosecond transient absorption spectroscopy (fs-TAS) was conducted using a commercial Helios setup from Ultrafast Systems with a Ti: sapphire regenerative amplified laser system (Coherent Libra) delivered laser pulses at 750 nm (100 fs, 1 kHz). An optical parametric amplifier (Vitara, Coherent) pumped by the regenerative amplifier was used to generate the pump beam at 750 nm (at wavelength resonant with the absorption of PSMA). The probe beam was generated by focusing part of the fundamental femtosecond laser beam onto a sapphire plate or Yttrium aluminum garnet plate for visible (Vis) and near-IR (NIR) spectral windows, respectively. TAS results in this work are presented in the unit of ΔOD, negative features can reflect ground-state bleaching (GSB) or stimulated emission (SE), a positive signal is an excited-state absorption (ESA). During TA measurements, the samples were kept in nitrogen to avoid photodegradation. The pump fluence was kept at <5 $\mu J/cm^2$ to minimize the exciton−exciton annihilation effect.

2. Device fabrication and measurements

Device fabrication: The patterned indium tin oxide (ITO, sheet resistance = 15 Ω $square^{-1}$) glass substrates were sequentially ultrasonicated with detergent, deionized water, acetone, and

isopropanol. Then, the ITO glasses were treated with UV-ozone for 30 min. Poly(3,4-ethylenedioxythiophene):polystyrene sulfonate (PEDOT:PSS) (Bay PVP. Al 4083, Bayer AG) was filtrated through a 0.45 μm nylon filter and then spin-coated on the cleaned ITO substrates at 5000 rpm for 60 s to form a thin layer (35 nm). For the layer-by-layer devices, the PM6 solution (11 mg/mL in chlorobenzene) was spin-coated on the ITO/PEDOT:PSS substrates at a speed of 3000 rpm for 30 s form a ~60 nm thickness of the polymer donor layer. Then the PSMA solutions (8 mg/mL in chloroform with 2 v% of 1-chloronaphthalene additive and 0.04 wt% of benzyl viologen dopant) were spin-coated on the ITO/PEDOT:PSS substrates at a speed of 3000 rpm for 30 s form a ~40 nm thickness of the electron acceptor layer. The substrates were then baked at 100 ^{o}C for 10 min. PNDIT-F3N solution (0.5 mg/mL in methanol with 5 v% of acetic acid) was spin-coated on the top of the active layer to form a thin cathode interlayer (~10 nm). Finally, argentum electrode (Ag, 100 nm) was deposited under high vacuum (~10^{-5} Pa) in an evaporation chamber. For the PM6:PSMA-based BHJ devices, PM6:PSMA solution (14.3 mg/mL in chloroform with 1v% of 1-chloronaphthalene additive and 0.04 wt% of benzyl viologen dopant) was spin-coated on the ITO/PEDOT:PSS substrates at a speed of 3000 rpm for 30 s form a ~100 nm thickness of the active layer. The other conditions kept identical to the layer-by-layer devices. For the D18-Cl/PSMA-based layer-by-layer devices, the D18-Cl solution (5.5 mg/mL in chloroform) was spin-coated on the ITO/PEDOT:PSS substrates at a speed of 3000 rpm for 30 s form a ~60 nm thickness of the polymer donor layer. The other conditions kept identical to the PM6-based layer-by-layer devices. The device area was exactly fixed at 4.00 mm^2. For the electron-only devices, a structure of ITO/ZnO/PSMA/PNDIT-F3N/Ag was employed. The ZnO layer was prepared by spin-coated the diethyl zinc solution (~0.2 M in tetrahydrofuran) in dry air and then baked at 180 ^{o}C for 30 min. The deposition for the other layers were same to the aforementioned device preparation procedure.

Device measurements: The *I-V* characterization was performed on a computer-controlled Keithley 2400 Source under AM1.5G (100 mW cm^{-2}) using a solar simulator (XES-70S1, SAN-EI), which was calibrated by a standard Si solar cell (AK-200, Konica Minolta, Inc.). The EQE values were measured with an EQ-R solar quantum efficiency test system (Enlitech Co., Ltd., Taiwan, China). All fabrication and characterization processes, except for the HTLs preparation and EQE measurements, were conducted in a high purity argon filled glove box. The transient photovoltage or photocurrent (TPV or TPC) decay measurements were obtained by using a pulsed double frequency Nd:YAG laser (Brio, 1000 Hz), at 550 nm with an ultra-low light intensity and a sub-nanosecond resolved digital oscilloscope (Tektronix DPO 7104).

The dynamics curves were recorded on a digital oscilloscope at a 50-Ω (short-circuit condition for TPC) resistor and a mega Ω (open-circuit condition for TPV).

3. Supplementary figures

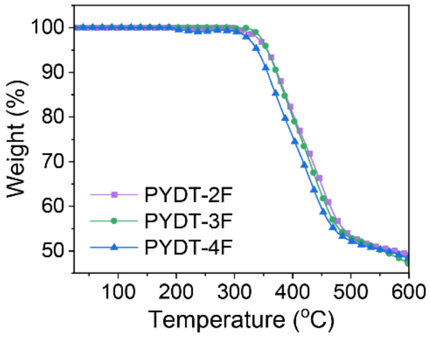

Figure S1 TGA curves of the PSMAs.

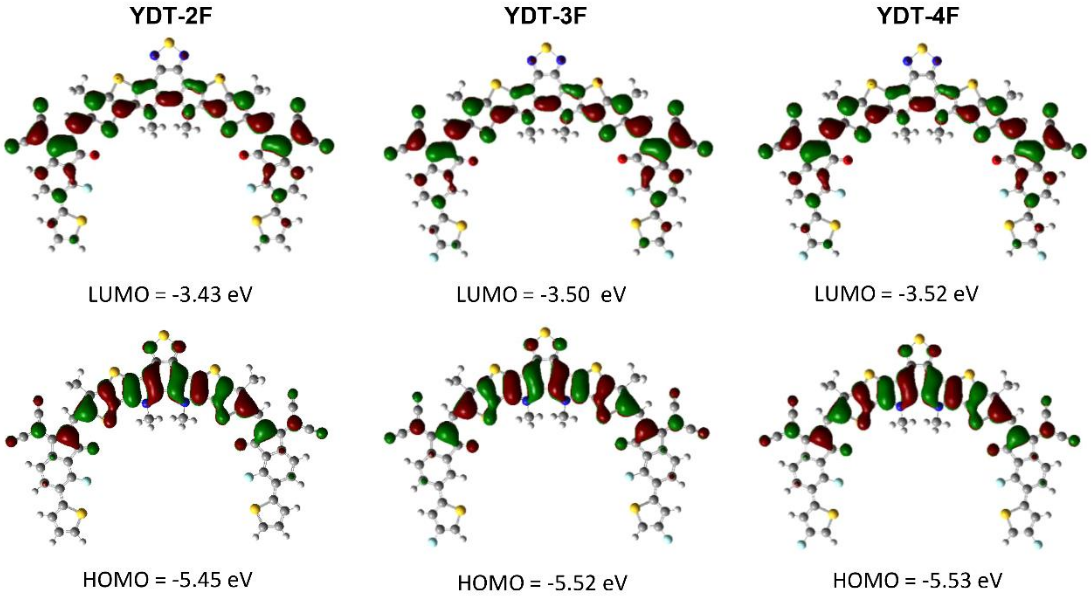

Figure S2. HOMO/LUMO levels of the monomers of YDT-2F, YDT-3F and YDT-4F obtained by DFT calculations.

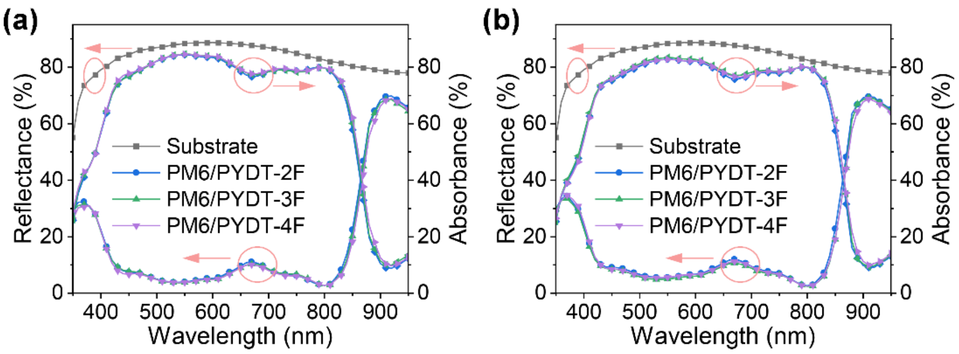

Figure S3. a) Reflectance of the substrate (all-PSCs excluded active layer) and all-PSC devices. b) Reflectance of the (all-PSCs excluded active layer) and all-PSC devices with BV doping.

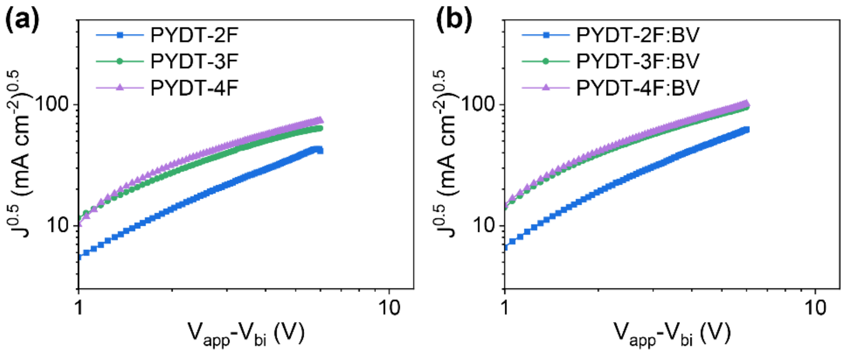

Figure S4. $J^{0.5}$-V curves of the electron-only device (a) without BV doping and (b) with BV doping.

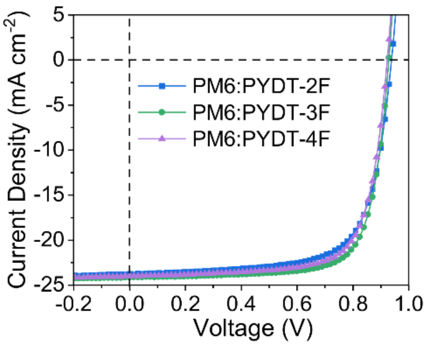

Figure S5. *J-V* curves the BHJ all-PSCs using PM6 donor and doped with 0.04 wt% BV.

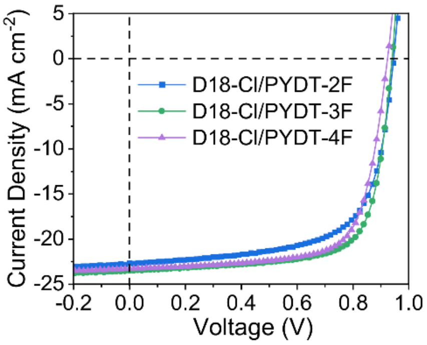

Figure S6. *J-V* curves the layer-by-layer processed all-PSCs using D18-Cl donor and doped with 0.04 wt% BV.

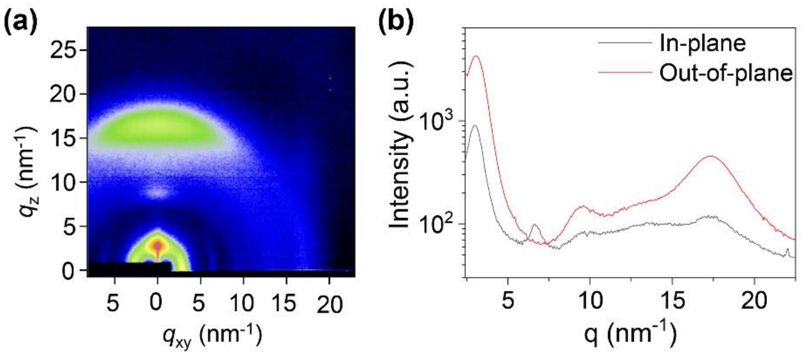

Figure S7. a) GIWAXS pattern of PM6 donor. b) The corresponding in-plane and out-of-plane line-cuts.

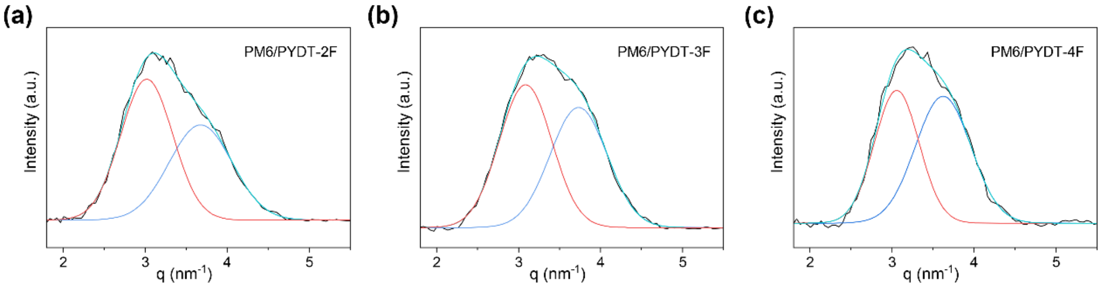

Figure S8. Peak fitting plots of the lamellar diffractions. a) PM6/PYDT-2F. b) PM6/PYDT-3F. c) PM6/PYDT-4F.

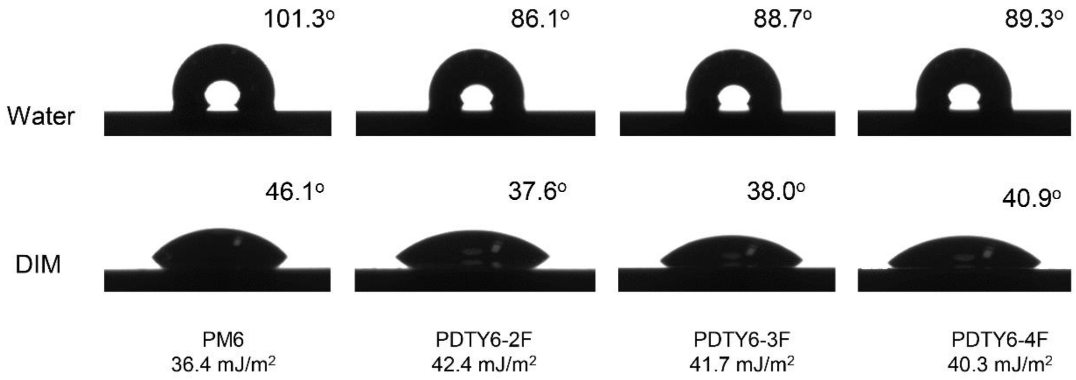

Figure S9. Water and diiodomethane (DIM) droplet contact angles of PM6 and the PSMAs.

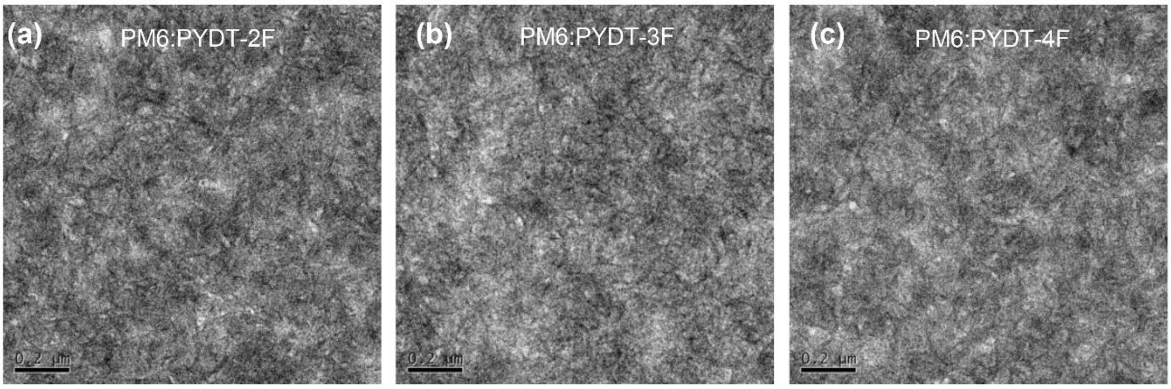

Figure S10. TEM images of the PM6:PSMA blend films.

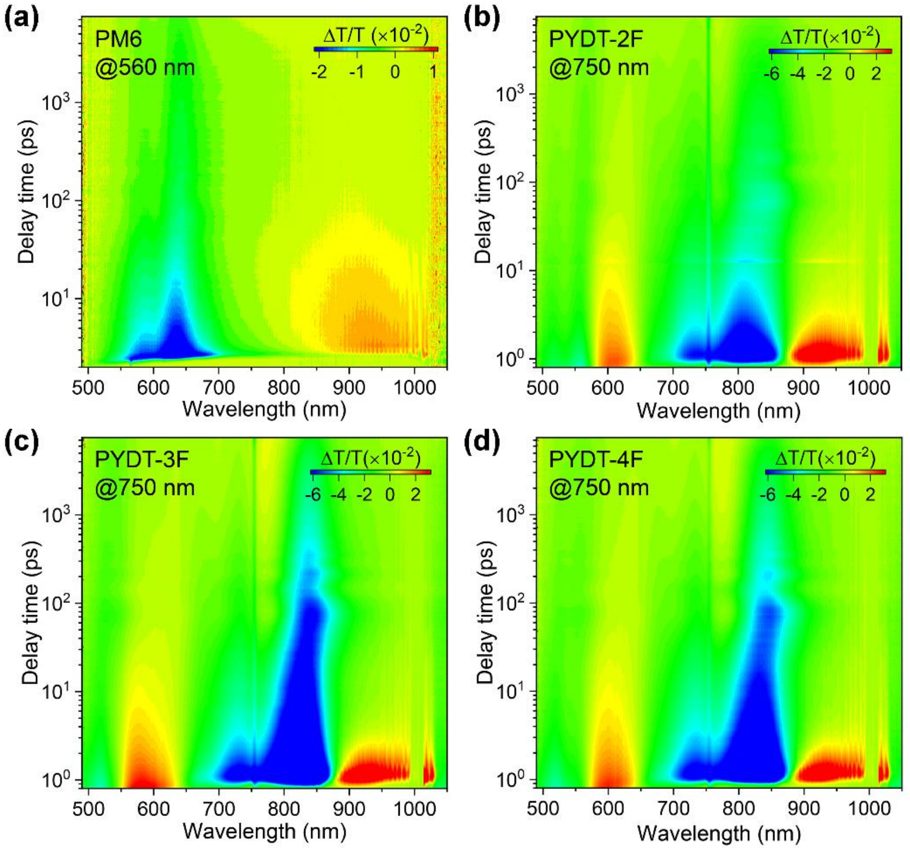

Figure S11. 2D fs-TAS images of the neat donor and acceptor films. a) PM6. b) PYDT-2F. c) PYDT-3F. d) PYDT-4F.

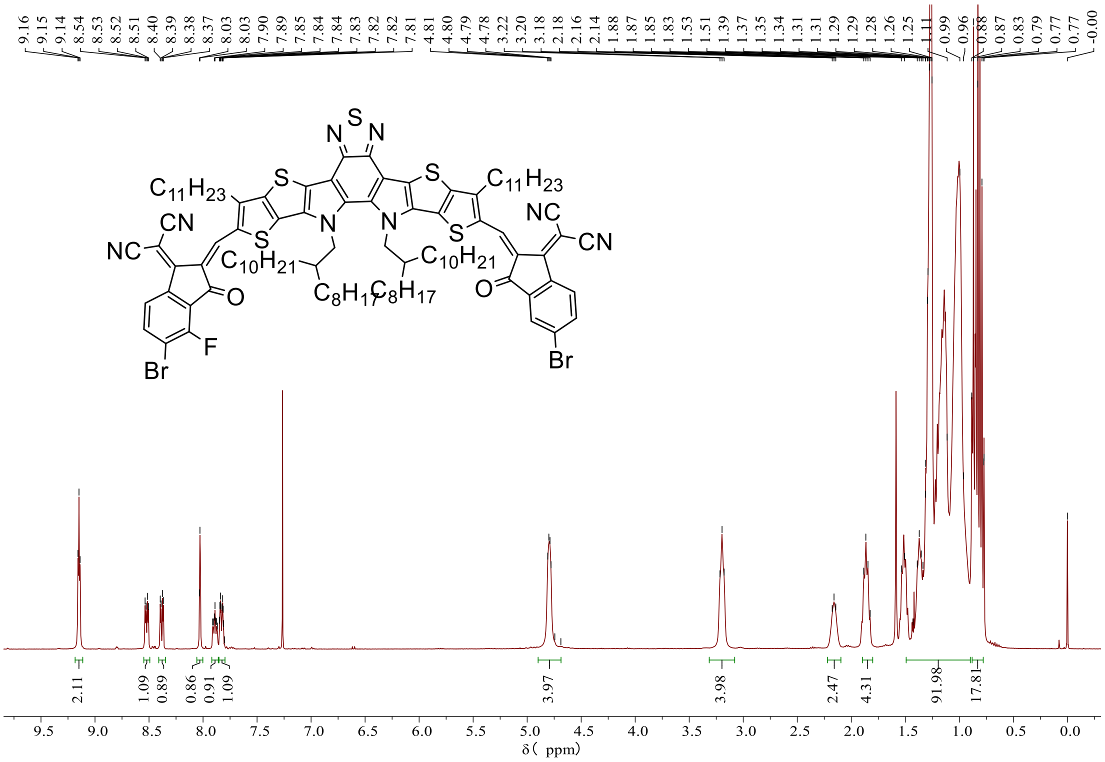

Figure S12. ^1H NMR spectrum of compound **3a** in $CDCl_3$.

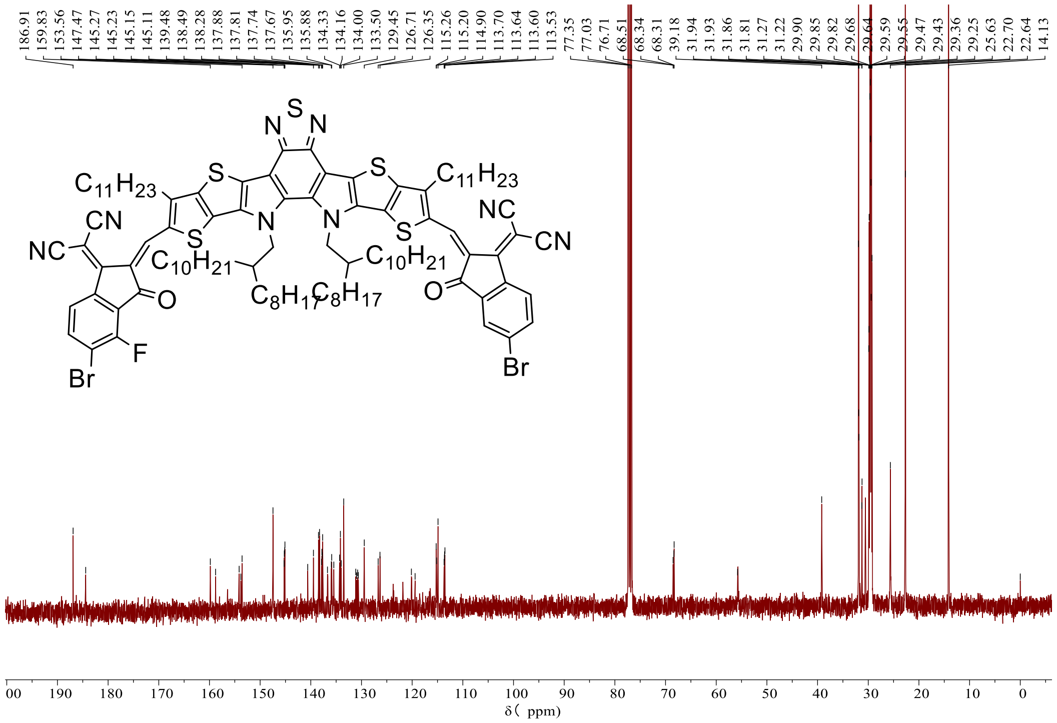

Figure S13. ^{13}C NMR spectrum of compound **3a** in $CDCl_3$.

4. Supplementary Tables

Table S1. Summarized photovoltaic parameters of the Y-series PSMAs with PCEs over 10%.

Active layer	V_{oc} [V]	J_{sc} [mA cm^{-2}]	FF [%]	PCE [%]	Reference
PBDBT:PTPBT-ET	0.899	21.33	65.3	12.52	[3]
PBDB-T:PF5-Y5	0.956	20.65	74.0	14.45	[4]
PBDB-T:PJ1-H	0.90	22.3	70	14.4	[5]
PM6:PY-IT	0.933	23.30	72.3	15.05	[6]
PM6:PY-IOT	0.939	19.71	65.6	12.12	[6]
PTzBI-oF:PFA1	0.87	23.96	72.67	15.11	[7]
PM6:L11	0.95	18.0	64.3	11.1	[8]
PM6:L14	0.96	20.6	72.1	14.3	[8]
PM6:L14	0.95	22.10	74.1	15.62	[9]
PM6:L14	0.953	21.12	71.60	14.41	[10]
PM6:L15	0.953	22.21	71.86	15.22	[10]
PM6:L15:MBTI	0.957	22.91	73.83	16.18	[10]
PBDB-T:A701	0.92	18.27	64	10.70	[11]
PM6:PYT$_L$	0.93	20.92	64.51	12.55	[1]

PM6:PYT$_M$	0.93	21.78	66.33	13.44	[1]
PM6:PYT1	0.938	21.50	66.66	13.43	[12]
PBDB-T:PYE20	0.905	20.97	71.63	13.60	[13]
PBDB-T:PG1	0.94	17.8	69	11.5	[14]
PBDB-T:PS-Se	0.874	23.27	68.0	13.83	[15]
PBDB-T:PN-Se	0.907	24.82	71.8	16.16	[15]
PBDB-T:PFY-0Se	0.904	20.9	68.8	13.0	[16]
PBDB-T:PFY-1Se	0.894	21.2	72.9	13.8	[16]
PBDB-T:PFY-2Se	0.875	23.4	72.0	14.7	[16]
PBDB-T:PFY-3Se	0.871	23.6	73.7	15.1	[16]
PBDB-T:PFY-2TS	0.906	20.47	66.3	12.31	[17]
PBDB-T:PFY-DTC	0.863	20.2	63.6	11.08	[17]
PM6:PYT-2S	0.941	22.3	70.7	14.8	[18]
PM6:PYT-1S1Se	0.926	24.1	73.0	16.3	[18]
PM6:PYT-2Se	0.908	23.9	71.4	15.5	[18]
PBDB-T:PYT	0.892	20.8	69.6	12.9	[19]
PBDB-T:PZT	0.909	23.2	68.6	14.5	[19]
PBDB-T:PZT-γ	0.896	24.7	71.3	15.8	[19]
PBDB-T:PYTS-0.3	0.92	22.91	70	14.68	[20]
PBDBT:PTPBT-ET0.1	0.888	23.15	66.31	13.63	[21]
PBDBT:PTQ10:PTPBT-ET0.1	0.909	23.68	67.64	14.56	[21]
JD40:PA-5	0.87	24.05	76.88	16.11	[22]
JD40:PA6-M	0.92	22.42	72.41	14.99	[22]
JD40:PJ1	0.91	23.2	75	15.8	[23]
JD40-S:PJ1	0.94	21.2	69	13.7	[23]
JD40-F:PJ1	0.97	18.7	65	11.8	[23]
PBDB-T:PTY6	0.86	20.64	68	12.2	[24]
PM6:PY-IT	0.937	21.90	73.6	15.11	[25]
PM6:PY-IT:BN-T	0.955	22.65	74.3	16.09	[25]
PBDB-T:Y5-Se-In	0.86	21.74	72	13.38	[26]
PBDB-T:Y5-BiSe-Out	0.92	18.12	66	10.67	[26]
PBDB-T:PYN-BDT	0.87	21.33	65	12.06	[27]
PBDB-T:PYN-BDTF	0.86	22.28	69	13.22	[27]
PBDB-T:RRd-C20	0.93	19.67	63	11.59	[28]
PBDB-T:RRd-C24	0.93	20.34	67	12.18	[28]
PBDB-T:RRg-C24	0.88	23.54	73	15.12	[28]
PBDB-T:RRg-C24	0.88	21.67	71	13.53	[28]
PM6:PYT	0.96	21.3	70.8	14.5	[29]
PM6:PY2F-T	0.87	24.2	71.2	15.0	[29]
PM6:PYT:PY2F-T	0.90	25.2	76.0	17.2	[29]

PM6: PBTIC-γ-2F2T	0.95	22.56	66.89	14.34	[30]
PM6: PBTIC-γ-2T	0.95	20.85	60.22	11.92	[30]
PBDB-T:PYTT-1	0.93	20.66	70.35	13.54	[31]
PBDB-T:PYTT-2	0.91	22.00	71.53	14.32	[31]
PBDB-T:PYTT-3	0.82	21.99	68.47	12.41	[31]
PM6:PBN25	0.89	22.62	71.6	14.36	[32]
PBDB-T: PYT	0.88	21.30	62.65	11.75	[33]
PBDB-T: PYT-TOE(10)	0.91	21.73	64.63	12.77	[33]
PBDB-T: PYT-TOE(20)	0.91	18.75	61.64	10.49	[33]
PBDB-T:PYT	0.887	22.64	70.02	14.06	[34]
PBDB-T/PYT	0.891	23.03	73.98	15.17	[34]
D18:SY6	0.86	21.81	69.98	13.13	[35]
D18:NC-PY6	0.87	20.66	68.42	12.30	[35]
PM6:PY-T	0.93	21.30	67.35	13.37	[36]
PM6:PY2F-T	0.86	24.27	72.62	15.22	[36]
PM6:PYF-T	0.891	23.1	68.0	14.0	[2]
PM6:PYF-T-o	0.901	23.3	72.4	15.2	[2]
PM6:PY-T	0.95	17.86	65.0	11.09	[37]
PM6:PYF-T	0.89	23.41	67.73	14.10	[37]
PBDB-T:PJ1	0.90	21.92	72.61	14.34	[37]
PBDB-T:PJ1:PJ2	0.91	21.46	20.39	72.98	[37]
JD40:PJTET	0.92	18.58	63.84	10.93	[38]
JD40:PJTVT	0.89	23.75	76.40	16.13	[38]
PBDB-TL_{LW}:PJ1	0.91	21.9	73.3	14.6	[39]
PBDB-TL_{MW}:PJ1	0.90	22.7	75.3	15.4	[39]
PBDB-TL_{HW}:PJ1	0.91	20.4	69.9	13.0	[39]
PBQx-Me-TF:PBTIC-γ-TT	0.903	20.13	59.77	10.86	[40]
PBQx-H-TF:PBTIC-γ-TT	0.916	22.88	67.80	14.21	[40]
PBDB-T:PYV	0.840	20.67	62.66	11.51	[41]
PBDB-T:PYV-Tz	0.886	21.73	67.62	13.02	[41]
PTzBI-oF:PS1	0.92	22.47	66.70	13.8	[42]
PM6:PY2S-H	0.941	22.3	70.7	14.8	[43]
PM6:PY2S-F	0.920	23.3	70.5	15.1	[43]
PM6:PY2Se-F	0.885	24.4	72.2	15.6	[43]
PM6:PY2Se-Cl	0.884	24.5	74.3	16.1	[43]
PTzBI-oF:PFA1	0.83	24.11	71.17	14.50	[44]
PM6:PY-DT	0.949	23.73	74.4	16.76	[45]
PM6:PY-V-γ	0.912	24.8	75.8	17.1	[46]
PM6:PY-T-γ	0.929	24.1	71.9	16.1	[46]
PM6:PY-2T-γ	0.933	23.5	69.9	15.3	[46]

PBDB-T/PYT	0.91	23.07	77	16.05	[47]
PM6/PYDT-2F	0.935	24.23	72.08	16.33	This work
PM6/PYDT-3F	0.923	24.61	77.01	17.49	This work
PM6/PYDT-4F	0.915	24.49	75.20	16.85	This work

Table S2. Photovoltaic parameters of the BHJ devices containing different PSMAs using PM6 as the polymer donor.

D:A	BV doping	V_{oc} [V]	J_{sc} [mA cm^{-2}]	FF [%]	PCE [%]
PM6:PYDT-2F	Yes	0.929	23.71	71.25	15.69 (15.31±0.27)[a)]
PM6:/PYDT-3F	Yes	0.920	24.15	75.01	16.67 (16.35±0.23)
PM6:PYDT-4F	Yes	0.913	24.02	73.82	16.19 (15.82±0.24)

[a)]Average PCEs with standard deviations were calculated from 10 individual devices.

Table S3. Photovoltaic parameters of the layer-by-layer processed devices containing different PSMAs using D18-Cl as the polymer donor.

D/A	BV doping	V_{oc} [V]	J_{sc} [mA cm^{-2}]	FF [%]	PCE [%]
D18-Cl/PYDT-2F	Yes	0.947	22.67	66.7	14.31 (13.87±0.37)[a)]
D18-Cl/PYDT-3F	Yes	0.940	23.51	72.7	16.07 (15.68±0.25)
D18-Cl/PYDT-4F	Yes	0.924	23.29	71.6	15.40 (15.10±0.26)

[a)]Average PCEs with standard deviations were calculated from 10 individual devices.

Table S4. Ordered molecular structure parameters of the pristine and blend films.

Film	Lamellar stacking (100)				π–π stacking (010)			
	Location [nm^{-1}]	d_l [Å]	FWHM [nm^{-1}]	CCL[a)] [Å]	Location [nm^{-1}]	d_π [Å]	FWHM [nm^{-1}]	CCL [Å]
PM6	3.01	20.9	0.930	60.8	17.4	3.61	2.71	23.2
PYDT-2F	3.72	16.9	1.12	56.1	17.2	3.65	3.54	16.0
PYDT-3F	3.74	16.8	0.94	60.1	17.2	3.65	2.84	19.9
PYDT-4F	3.77	16.7	0.88	64.5	17.2	3.65	2.65	21.3
PM6/PYDT-2F	3.01<br>3.69	20.9<br>17.0	0.74<br>0.90	76.4<br>62.8	17.2	3.65	2.93	19.3
PM6/PYDT-3F	3.08<br>3.73	20.4<br>16.8	0.72<br>0.81	78.5<br>69.8	17.4	3.61	2.72	20.8
PM6/PYDT-4F	3.10<br>3.73	20.3<br>16.8	0.70<br>0.79	80.7<br>71.5	17.4	3.61	2.69	21.0

[a)] CCL = 0.9*2π/FWHM

5. References

[1] W. Wang, Q. Wu, R. Sun, J. Guo, Y. Wu, M. M. Shi, W. Y. Yang, H. N. Li, J. Min, *Joule* **2020**, *4*, 1070.

[2] H. Yu, M. Pan, R. Sun, I. Agunawela, J. Zhang, Y. Li, Z. Qi, H. Han, X. Zou, W. Zhou, S. Chen, J. Y. L. Lai, S. Luo, Z. Luo, D. Zhao, X. Lu, H. Ade, F. Huang, J. Min, H. Yan, *Angew. Chem. Int. Ed.* **2021**, *60*, 10137.

[3] J. Du, K. Hu, L. Meng, I. Angunawela, J. Zhang, S. Qin, A. Liebman-Pelaez, C. Zhu, Z. Zhang, H. Ade, Y. Li, *Angew. Chem. Int. Ed.* **2020**, *59*, 15181.

[4] Q. Fan, Q. An, Y. Lin, Y. Xia, Q. Li, M. Zhang, W. Su, W. Peng, C. Zhang, F. Liu, L. Hou, W. Zhu, D. Yu, M. Xiao, E. Moons, F. Zhang, T. D. Anthopoulos, O. Inganäs, E. Wang, *Energy Environ. Sci.* **2020**, *13*, 5017.

[5] T. Jia, J. Zhang, W. Zhong, Y. Liang, K. Zhang, S. Dong, L. Ying, F. Liu, X. Wang, F. Huang, Y. Cao, *Nano Energy* **2020**, *72*, 104718.

[6] Z. Luo, T. Liu, R. Ma, Y. Xiao, L. Zhan, G. Zhang, H. Sun, F. Ni, G. Chai, J. Wang, C. Zhong, Y. Zou, X. Guo, X. Lu, H. Chen, H. Yan, C. Yang, *Adv. Mater.* **2020**, *32*, 2005942.

[7] F. Peng, K. An, W. Zhong, Z. Li, L. Ying, N. Li, Z. Huang, C. Zhu, B. Fan, F. Huang, Y. Cao, *ACS Energy Lett.* **2020**, *5*, 3702.

[8] H. Sun, H. Yu, Y. Shi, J. Yu, Z. Peng, X. Zhang, B. Liu, J. Wang, R. Singh, J. Lee, Y. Li, Z. Wei, Q. Liao, Z. Kan, L. Ye, H. Yan, F. Gao, X. Guo, *Adv. Mater.* **2020**, *32*, 2004183.

[9] B. Liu, H. L. Sun, J. W. Lee, J. Yang, J. W. Wang, Y. C. Li, B. B. Li, M. Xu, Q. G. Liao, W. Zhang, D. X. Han, L. Niu, H. Meng, B. J. Kim, X. G. Guo, *Energy Environ. Sci.* **2021**, *14*, 4499.

[10] H. Sun, B. Liu, Y. Ma, J. W. Lee, J. Yang, J. Wang, Y. Li, B. Li, K. Feng, Y. Shi, B. Zhang, D. Han, H. Meng, L. Niu, B. J. Kim, Q. Zheng, X. Guo, *Adv. Mater.* **2021**, *33*, 2102635.

[11] A. Tang, J. Li, B. Zhang, J. Peng, E. Zhou, *ACS Macro Lett.* **2020**, *9*, 706.

[12] Q. Wu, W. Wang, T. Wang, R. Sun, J. Guo, Y. Wu, X. Jiao, C. J. Brabec, Y. Li, J. Min, *Sci. China Chem.* **2020**, *63*, 1449.

[13] Y. Wu, Q. Wu, W. Wang, R. Sun, J. Min, *Sol. RRL* **2020**, *4*, 2000409.

[14] Z. Yin, Y. Wang, Q. Guo, L. Zhu, H. Liu, J. Fang, X. Guo, F. Liu, Z. Tang, M. Zhang, Y. Li, *J. Mater. Chem. C* **2020**, *8*, 16180.

[15] J. Du, K. Hu, J. Zhang, L. Meng, J. Yue, I. Angunawela, H. Yan, S. Qin, X. Kong, Z. Zhang, B. Guan, H. Ade, Y. Li, *Nat. Commun.* **2021**, *12*, 5264.

[16] Q. Fan, H. Fu, Q. Wu, Z. Wu, F. Lin, Z. Zhu, J. Min, H. Y. Woo, A. K. Jen, *Angew. Chem. Int. Ed.* **2021**, *60*, 15935.

[17] Q. Fan, R. Ma, T. Liu, J. Yu, Y. Xiao, W. Su, G. Cai, Y. Li, W. Peng, T. Guo, Z. Luo, H. Sun, L. Hou, W. Zhu, X. Lu, F. Gao, E. Moons, D. Yu, H. Yan, E. Wang, *Sci. China Chem.* **2021**, *64*, 1380.

[18] H. Fu, Q. Fan, W. Gao, J. Oh, Y. Li, F. Lin, F. Qi, C. Yang, T. J. Marks, A. K. Y. Jen, *Sci. China Chem.* **2021**, *65*, 309.

[19] H. Fu, Y. Li, J. Yu, Z. Wu, Q. Fan, F. Lin, H. Y. Woo, F. Gao, Z. Zhu, A. K. Jen, *J. Am. Chem. Soc.* **2021**, *143*, 2665.

[20] Z. Genene, J. W. Lee, S. W. Lee, Q. Chen, Z. Tan, B. A. Abdulahi, D. Yu, T. S. Kim, B. J. Kim, E. Wang, *Adv. Mater.* **2022**, *34*, 2107361.

[21] K. Hu, J. Du, C. Sun, C. Zhu, J. Zhang, J. Yao, Z. Zhang, Y. Wan, Z. Zhang, L. Meng, Y. Li, *Energ. Fuel.* **2021**, *35*, 19045.

[22] J. Jia, Q. Huang, T. Jia, K. Zhang, J. Zhang, J. Miao, F. Huang, C. Yang, *Adv. Energy Mater.* **2021**, *12*, 2103193.

[23] T. Jia, J. Zhang, K. Zhang, H. Tang, S. Dong, C.-H. Tan, X. Wang, F. Huang, *J. Mater. Chem. A* **2021**, *9*, 8975.

[24] S. Li, X. Yuan, Q. Zhang, B. Li, Y. Li, J. Sun, Y. Feng, X. Zhang, Z. Wu, H. Wei, M. Wang, Y. Hu, Y. Zhang, H. Y. Woo, J. Yuan, W. Ma, *Adv. Mater.* **2021**, *33*, 2101295.

[25] T. Liu, T. Yang, R. Ma, L. Zhan, Z. Luo, G. Zhang, Y. Li, K. Gao, Y. Xiao, J. Yu, X. Zou, H. Sun, M. Zhang, T. A. Dela Peña, Z. Xing, H. Liu, X. Li, G. Li, J. Huang, C. Duan, K. S. Wong, X. Lu, X. Guo, F. Gao, H. Chen, F. Huang, Y. Li, Y. Li, Y. Cao, B. Tang, H. Yan, *Joule* **2021**, *5*, 914.

[26] S. Seo, C. Sun, J. W. Lee, S. Lee, D. Lee, C. Wang, T. N. L. Phan, G. U. Kim, S. Cho, Y. H. Kim, B. J. Kim, *Adv. Funct. Mater.* **2021**, 10.1002/adfm.202108508.

[27] N. Su, R. Ma, G. Li, T. Liu, L.-W. Feng, C. Lin, J. Chen, J. Song, Y. Xiao, J. Qu, X. Lu, V. K. Sangwan, M. C. Hersam, H. Yan, A. Facchetti, T. J. Marks, *ACS Energy Lett.* **2021**, *6*, 728.

[28] C. Sun, J. W. Lee, S. Seo, S. Lee, C. Wang, H. Li, Z. Tan, S. K. Kwon, B. J. Kim, Y. H. Kim, *Adv. Energy Mater.* **2021**, *12*, 2103239.

[29] R. Sun, W. Wang, H. Yu, Z. Chen, X. Xia, H. Shen, J. Guo, M. Shi, Y. Zheng, Y. Wu, W. Yang, T. Wang, Q. Wu, Y. Yang, X. Lu, J. Xia, C. J. Brabec, H. Yan, Y. Li, J. Min, *Joule* **2021**, *5*, 1548.

[30] H. T. Wang, H. Chen, W. C. Xie, H. J. Lai, T. X. Zhao, Y. L. Zhu, L. Chen, C. X. Ke, N. Zheng, F. He, *Adv. Funct. Mater.* **2021**, *31*, 2100877.

[31] T. Wang, R. Sun, W. Wang, H. Li, Y. Wu, J. Min, *Chem. Mater.* **2021**, *33*, 761.

[32] Y. Wang, N. Wang, Q. Yang, J. Zhang, J. Liu, L. Wang, *J. Mater. Chem. A* **2021**, *9*, 21071.

[33] F. Wu, J. Liu, J. Liu, J. Oh, B. Huang, D. Chen, Z. Liu, Q. He, C. Yang, L. Chen, *ChemElectroChem* **2021**, *8*, 3936.

[34] Q. Wu, W. Wang, Y. Wu, Z. Chen, J. Guo, R. Sun, J. Guo, Y. Yang, J. Min, *Adv. Funct. Mater.* **2021**, *31*, 2010411.

[35] X. M. Wu, X. Y. Hao, P. Deng, H. P. Chen, *Dyes Pigments* **2021**, *196*, 109824.

[36] H. Yu, S. Luo, R. Sun, I. Angunawela, Z. Qi, Z. Peng, W. Zhou, H. Han, R. Wei, M. Pan, A. M. H. Cheung, D. Zhao, J. Zhang, H. Ade, J. Min, H. Yan, *Adv. Funct. Mater.* **2021**, *31*, 2100791.

[37] H. Yu, Z. Qi, J. Yu, Y. Xiao, R. Sun, Z. Luo, A. M. H. Cheung, J. Zhang, H. Sun, W. Zhou, S. Chen, X. Guo, X. Lu, F. Gao, J. Min, H. Yan, *Adv. Energy Mater.* **2021**, *11*, 2003171.

[38] J. Zhang, C. H. Tan, K. Zhang, T. Jia, Y. Cui, W. Deng, X. Liao, H. Wu, Q. Xu, F. Huang, Y. Cao, *Adv. Energy Mater.* **2021**, *11*, 2102559.

[39] L. Zhang, T. Jia, L. Pan, B. Wu, Z. Wang, K. Gao, F. Liu, C. Duan, F. Huang, Y. Cao, *Sci. China Chem.* **2021**, *64*, 408.

[40] T. Zhao, C. Cao, H. Wang, X. Shen, H. Lai, Y. Zhu, H. Chen, L. Han, T. Rehman, F. He, *Macromolecules* **2021**, *54*, 11468.

[41] X. Zhao, T. Wang, W. Wang, R. Sun, Q. Wu, H. Shen, J. Xia, Y. Wang, M. Zhang, J. Min, *Polymer* **2021**, *230*, 124104.

[42] C. Zhu, Z. Li, W. Zhong, F. Peng, Z. Zeng, L. Ying, F. Huang, Y. Cao, *Chem. Commun.* **2021**, *57*, 935.

[43] Q. Fan, H. Fu, Z. Luo, J. Oh, B. Fan, F. Lin, C. Yang, A. K. Y. Jen, *Nano Energy* **2022**, *92*, 106718.

[44] Z. Li, F. Peng, H. Quan, X. Qian, L. Ying, Y. Cao, *Chem. Eng. J.* **2022**, *430*, 132711.

[45] Y. Li, J. Song, Y. Dong, H. Jin, J. Xin, S. Wang, Y. Cai, L. Jiang, W. Ma, Z. Tang, Y. Sun, *Adv. Mater.* **2022**, *34*, 2110155.

[46] H. Yu, Y. Wang, H. K. Kim, X. Wu, Y. Li, Z. Yao, M. Pan, X. Zou, J. Zhang, S. Chen, D. Zhao, F. Huang, X. Lu, Z. Zhu, H. Yan, *Adv. Mater.* **2022**, *34*, 2200361.

[47] Y. Zhang, B. Wu, Y. He, W. Deng, J. Li, J. Li, N. Qiao, Y. Xing, X. Yuan, N. Li, C. J. Brabec, H. Wu, G. Lu, C. Duan, F. Huang, Y. Cao, *Nano Energy* **2022**, *93*, 106858.
